# Monozygotic twins concordant for Kleine-Levin syndrome

**DOI:** 10.1186/1471-2377-12-31

**Published:** 2012-05-30

**Authors:** Taro Ueno, Akira Fukuhara, Azusa Ikegami, Fumihiro Ohishi, Kazuhiko Kume

**Affiliations:** 1 Institute of Molecular Embryology and Genetics, Kumamoto University, Honjo, Kumamoto, 860-0811, Japan; 2 Sleep Center, Kuwamizu Hospital, Kuwamizu, Kumamoto, 862-0954, Japan

**Keywords:** Recurrent hypersomnia, Kleine-Levin syndrome, Monozygotic twins, HLA typing, Polysomnography, Actimetry

## Abstract

**Background:**

Kleine-Levin syndrome is a rare sleep disorder of unknown etiology. It is characterized by intermittent periods of excessive sleepiness, cognitive disturbances and behavioral abnormalities. Nine cases of familial Kleine-Levin syndrome have been identified, but there are no reported cases describing twins that are affected by the syndrome.

**Case presentation:**

We report the cases of 16-year-old monozygotic twin boys who both suffered from Kleine-Levin syndrome. In both cases, the onset of the first episode was preceded by an influenza infection. During symptomatic periods they slept for the entire day except for meals and bathroom visits. Actimetry recordings revealed that during symptomatic periods, daily activity was lower than that of asymptomatic periods, on the other hand, activity during the night was significantly higher in symptomatic periods than asymptomatic periods. Polysomnography (PSG) data during symptomatic periods revealed a decrease in sleep efficiency. Human leukocyte antigen (HLA) typing revealed no DQB1*02 loci. They were administered lithium carbonate but the beneficial effect was limited.

**Conclusions:**

Our observations suggest that Kleine-Levin syndrome may be due to genetic and autoimmune processes, although etiologic relationship to specific HLA type remains controversial.

## Background

Kleine-Levin syndrome is a rare idiopathic form of episodic hypersomnia that typically occurs during adolescence. The cardinal clinical features are recurrent hypersomnia, accompanied by cognitive disturbances and behavioral abnormalities [1]. The most typical form of classical Kleine-Levin syndrome is associated with hyperphagia [2,3], although hyperphagia is now optional after change of the criteria. Hypersexuality, behavioral disinhibition, delusions, autonomic alteration and hallucinations have also been described, but the patients show normal cognitive function and behavior between attacks. The pathogenesis of Kleine-Levin syndrome is not yet known. Although most cases of recurrent hypersomnia are sporadic, the occurrence of nine familial cases indicate that there may be a genetic predisposition to the syndrome [4-8] However, no cases of twins affected with Kleine-Levin syndrome have been reported [9]. In this case study we describe monozygotic twins suffering from the syndrome. This is the first case report describing twins affected with Kleine-Levin syndrome thereby supporting the theory that there is an underlying genetic predisposition to the syndrome.

## Case presentation

### Case 1

A 15-year-old boy visited our hospital complaining of recurrent episodes of hypersomnia. His first attack of hypersomnia started at the age of 13, one month after he had been treated with zanamivir for an influenza infection. The episodes of hypersomnia occurred once a month and each lasted for 7 to 10 days. During the symptomatic periods, the patient slept for the entire day except for meals and bathroom visits. He did not exhibit compulsive eating but instead ate less than usual. When questioned, he gave no answer and he later reported that it was difficult to understand what he was told. During the episode, he experienced visual hallucinations such as a fireball. Often, the attacks were immediately preceded by flu-like symptoms. Both of the twins are active baseball players in the middle school team, but both of them did not want to play during and immediately after the episodes. A physical examination of the patient was unremarkable. Between spells, he appeared animated and displayed normal social behavior.

**Figure 1:**
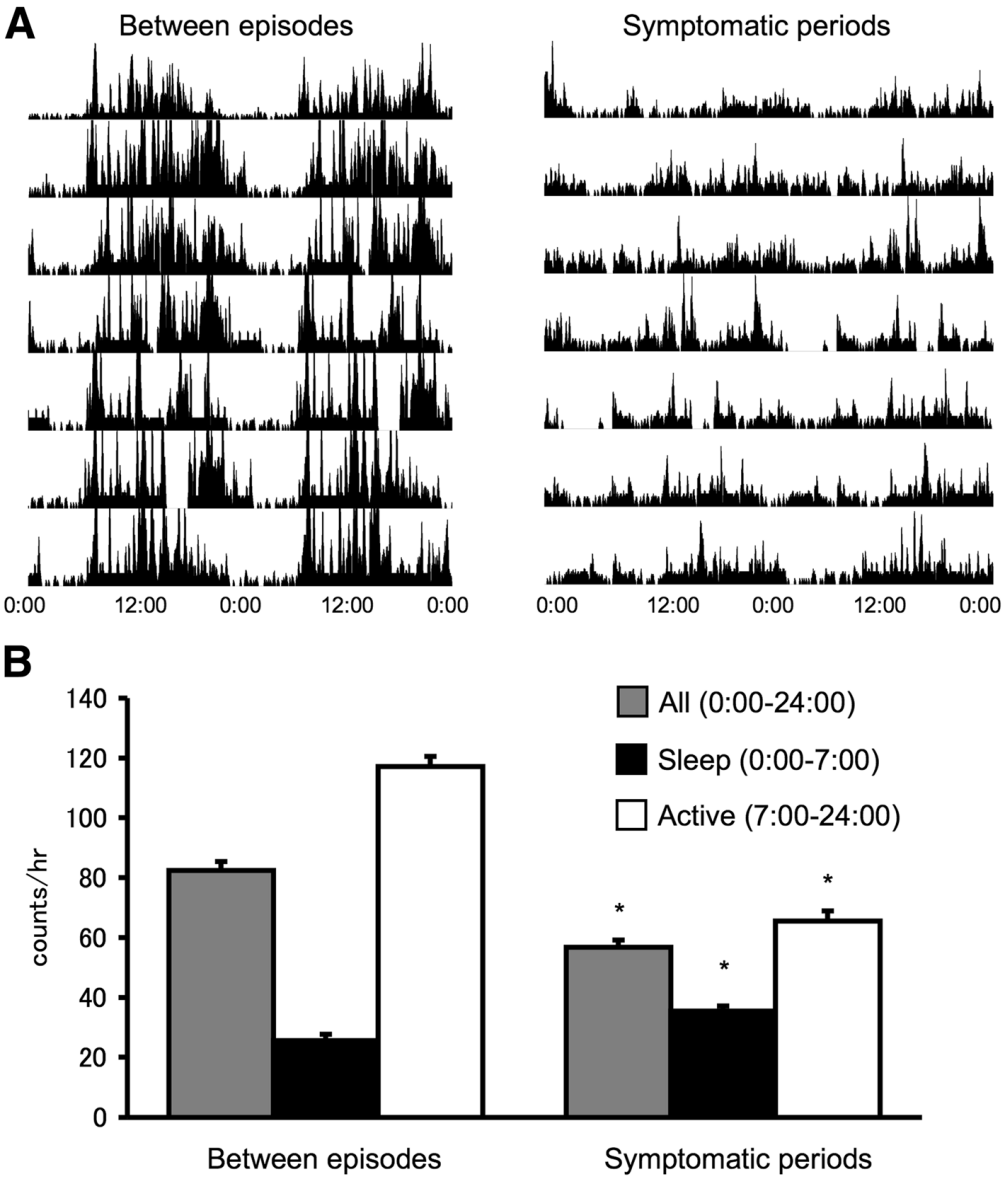
**Locomotor activity recorded using actimetry.(A)** Actogram records graphed as a double plot showing the locomotor activity of case 1. The actograms show the activity for the period between episodes and that of symptomatic periods. **(B)** Statistical analysis of locomotor activity. Based on bed time of the period between episodes, daily activity (0:00–24:00) was divided into sleep (0:00–7:00) and active (7:00–24:00) times. Data are represented as the mean ± SEM. Asterisks indicate statistically significant differences as determined by a Student’s *t*-test (p < 0.0005).

**Figure 2:**
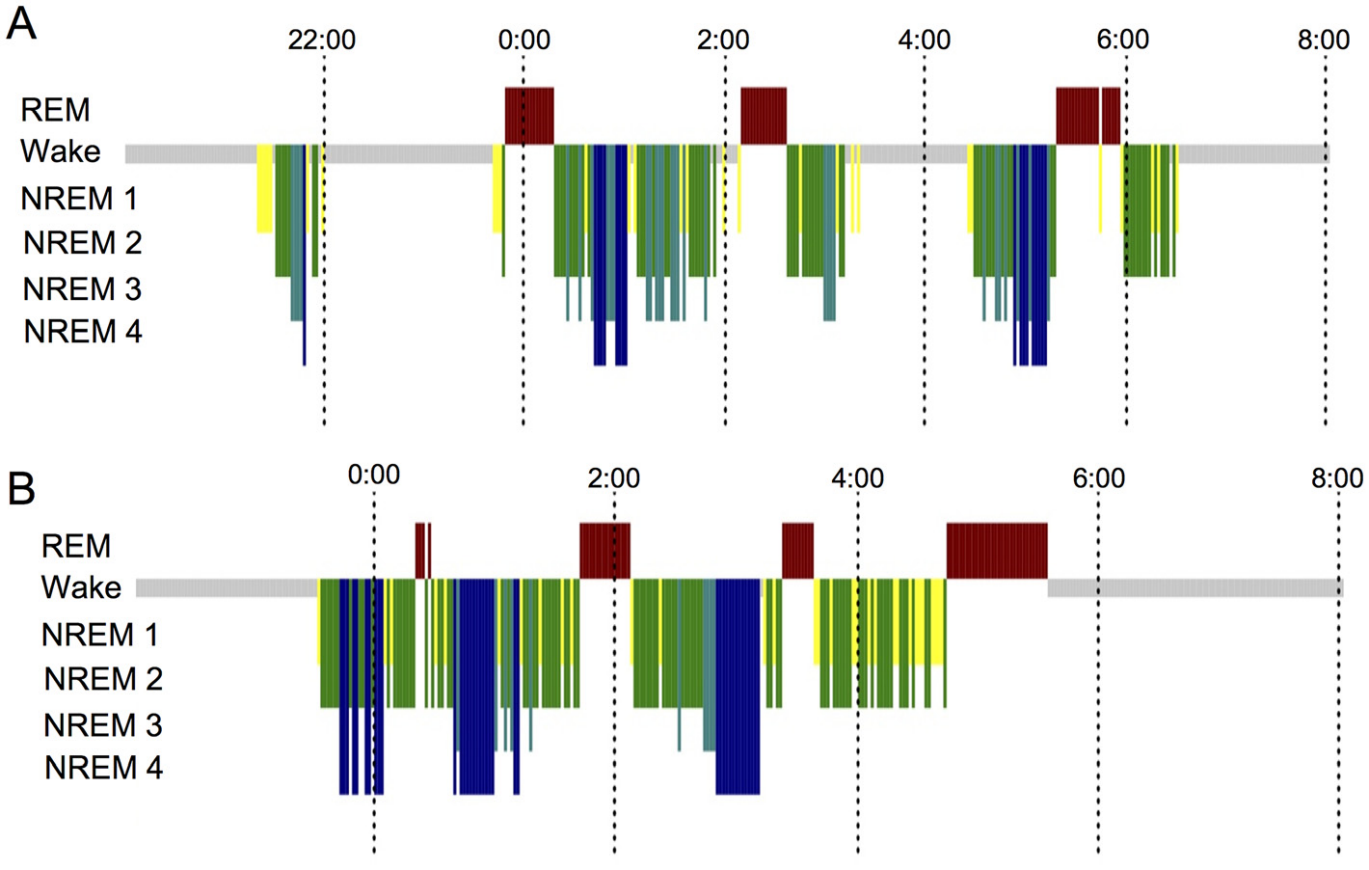
**Hypnograms of the twin boys.(A)** Hypnograms of case #1 during symptomatic period. **(B)** Hypnograms of case #2 during asymptomatic period.

Actimetry recording showed that during attacks, daily activity was lower than that of asymptomatic periods (Figure 1). On the other hand, activity during the night was significantly higher than in symptomatic periods. PSG was performed during a symptomatic night and revealed a decrease in sleep efficiency (63.6%, Table 1). In addition, frequent awakenings were observed during the nocturnal PSG (Figure 2A). HLA typing revealed the presence of the DQB1*0302/0601, DRB1*0407/1502 allele.

**Table 1:** **Analysis of sleep architecture during symptomatic period (case #1) and asymptomatic period (case #2)**

	**Case #1**	**Case #2**
Stage minutes (%)	symptomatic period	asymptomatic period
Wake	207 (36.4)	13.5 (3.6)
Stage 1	55 (9.7)	37 (10)
Stage 2	130 (22.8)	150 (40.5)
Stage 3	45 (7.9)	21 (5.7)
Stage 4	39 (6.9)	53 (14.3)
REM	94 (16.5)	98 (26.4)
Sleep latency	15	7
Sleep efficiency	63.6%	96.4%
Total bed time	569	370

Medication using lithium carbonate was initiated and gradually increased to 1000 mg/day with drug monitoring to keep the concentration within therapeutic window (0.8 – 1.2 mEq/l), and it reduced the frequency of the spells. As of November 2011, he has experienced 23 episodes.

### Case 2

The monozygotic twin brother of the individual described in case 1 suffered from repeated hypersomnolent spells from the age of 14. The first episode was preceded by an influenza infection that was treated with zanamivir. During episodes, he remained in bed either sleeping or physically inactive. His symptoms lasted for five to seven days, with intervals of three months between spells. He did not exhibit compulsive eating, but he preferred unusually (for him) sour foods. He had difficulty with verbal communication but could write down an answer when questioned. This speech abnormality became less prominent with subsequent spells. Attacks were immediately preceded by a flu-like illness. Between spells, he displayed no psychiatric disorder and maintained normal social and intellectual functions.

Actimetry recording showed that during attacks, activity during the day was lower than that of asymptomatic periods, on the other hand, the activity during night was significantly higher in symptomatic periods. PSG was performed during an asymptomatic night but revealed no significant findings (Table 1, Figure 2B). HLA typing revealed the presence of the DQB1*0302/0601, DRB1*0407/1502 allele.

Medication using lithium carbonate was initiated and gradually increased to 600 mg/day with drug monitoring, but it neither prevented nor reduced the duration of the spells. As of November 2011, he has experienced 10 episodes.

## Conclusions and discussion

Sleep disorders involve genetic susceptibility, environmental effects, and interactions between these factors [10]. The heritability of sleep patterns has been reported previously in studies of monozygotic twins, which may assist in the identification of genes involved in sleep disorders [11]. An understanding of sleep regulation at the molecular level is essential in the identification of targets for the treatment of sleep disorders. Although several familial cases of Kleine-Levin syndrome were reported [4-8], no cases of twins affected with the disease reported [9]. The cases of Kleine-Levin syndrome reported here are exceptional as they occur in monozygotic twins. For both patients the age of onset was similar and the symptoms and clinical course were typical. Actimetry demonstrated increased nocturnal activity and decreased daytime activity during symptomatic periods. PSG revealed low sleep efficiency during attacks, a decreased amount of SWS, sleep onset REM periods and sleep fragmentation. These PSG data are consistent with a previous report [12]. Despite the reduced amplitude in the circadian rhythm of activity, a previous endocrinological study reported no link between an underlying circadian disorder and recurrent hypersomnia [13]. Hypoperfusion [14] or hyperperfusion [15] of thalamus have been reported during the symptomatic period of Kleine-Levin syndrome with Tc-99 m ECD single photon emission tomography (SPECT) which suggest that there is an involvement of this brain tissue in the acute clinical presentation. Based on the generally young age of onset, the recurrence of symptoms and the frequent infectious trigger, an autoimmune etiology for Kleine-Levin syndrome has been suggested. In terms of genetic susceptibility, it has been suggested that the gene polymorphism of HLA-DQB1 is associated with recurrent hypersomnia [16]. However, in a more recent study using a larger independent sample, HLA DR and DQ alleles did not differ between cases and control subjects [17]. Both of the subjects in the case study reported here had been treated for an influenza infection at the onset of the first episode, but we did not identify DQB1*02. As for narcolepsy, which is a representative primary hypersomnia and has an established linkage with HLA subtype (HLA-DR2, DQB1*0602), increased onset following 2009 H1N1 winter influenza pandemic were reported recently [18]. Association with HLA is now controversial but the involvement of an autoimmune process remains plausible. Genome-wide mapping studies should be performed using the known familial cases to search for potential linkage. Finally, while this manuscript was under review, another monozygotic twin pair concordant for Kleine-Levin syndrome is reported [19]. Interestingly, they also found DQB1*0302/*0601 allele in the twins.

## Consent

Written informed consent was obtained from the patients for publication of this case report and any accompanying images. A copy of the written consent is available for review by the Editor-in-Chief of this journal.

## Abbreviations

PSG: Polysomnography; HLA: Human leukocyte antigen; REM: Rapid eye movement; SWS: Slow wave sleep; SPECT: Single photon emission tomography.

## Competing interests

The authors declare that they have no competing interest.

## Author’s contributions

TU and KK drafted the first manuscript and made a contribution to acquisition and interpretation of data. AF, AI, and FO provided technical oversight to the study, which included reviewing writing parts of the manuscript. All authors read and approved the final manuscript.

## Author’s information

Kuwamizu Hospital is an accredited hospital for sleep medicine certified by the Japanese Society of Sleep Research (JSSR). As of 2011, there are 71 of those hospitals in Japan, and there is only one in Kumamoto Prefecture, which has 1.8 million people. AI and KK are accredited specialist physicians for sleep medicine certified by JSSR. As of 2011, there are 381 accredited in Japan.

## Pre-publication history

The pre-publication history for this paper can be accessed here:

http://www.biomedcentral.com/1471-2377/12/31/prepub

## References

[B1] American Academy of Sleep Medicine The international classification of sleep disorders: diagnostic and coding manual 2005 2 Westchester, Ill: American Academy of Sleep Medicine

[B2] Kleine DW Periodische Schlafsucht Eur Neurol 1925 57 5–6 285 304

[B3] Levin M Narcolepsy (Gelineau’s syndrome) and other varieties of morbid somnolence Arch Neurol Psychiatry 1929 22 6 1172 1200 10.1001/archneurpsyc.1929.02220060069006

[B4] Poppe M Friebel D Reuner U Todt H Koch R Heubner G The Kleine-Levin syndrome - effects of treatment with lithium Neuropediatrics 2003 34 3 113 119 12910433 10.1055/s-2003-41273

[B5] BaHammam AS GadElRab MO Owais SM Alswat K Hamam KD Clinical characteristics and HLA typing of a family with Kleine-Levin syndrome Sleep Med 2008 9 5 575 578 10.1016/j.sleep.2007.06.015 17761456

[B6] Katz JD Ropper AH Familial Kleine-Levin syndrome: two siblings with unusually long hypersomnic spells Arch Neurol 2002 59 12 1959 1961 10.1001/archneur.59.12.1959 12470186

[B7] Rocamora R Gil-Nagel A Franch O Vela-Bueno A Familial recurrent hypersomnia: two siblings with Kleine-Levin syndrome and menstrual-related hypersomnia J Child Neurol 2010 25 11 1408 1410 10.1177/0883073810366599 20404354

[B8] Suwa K Toru M A case of periodic somnolence whose sleep was induced by glucose Psychiatry Clin Neurosci 1969 23 4 253 262 10.1111/j.1440-1819.1969.tb02878.x 5396056

[B9] Billiard M Jaussent I Dauvilliers Y Besset A Recurrent hypersomnia: a review of 339 cases Sleep Med Rev 2011 10.1016/j.smrv.2010.08.001 20970360

[B10] Tafti M Genetic aspects of normal and disturbed sleep Sleep Med 2009 10 Suppl 1 S17 21 19660984 10.1016/j.sleep.2009.07.002

[B11] Linkowski P EEG sleep patterns in twins J Sleep Res 1999 8 Suppl 1 11 13 10389101 10.1046/j.1365-2869.1999.00002.x

[B12] Huang YS Lin YH Guilleminault C Polysomnography in Kleine-Levin syndrome Neurology 2008 70 10 795 801 10.1212/01.wnl.0000304133.00875.2b 18316691

[B13] Mayer G Leonhard E Krieg J Meier-Ewert K Endocrinological and polysomnographic findings in Kleine-Levin syndrome: no evidence for hypothalamic and circadian dysfunction Sleep 1998 21 3 278 284 9595606 10.1093/sleep/21.3.278

[B14] Huang YS Guilleminault C Kao PF Liu FY SPECT findings in the Kleine-Levin syndrome Sleep 2005 28 8 955 960 16218078 10.1093/sleep/28.8.955

[B15] Itokawa K Fukui M Ninomiya M Yamamoto T Imabayashi E Tamura N Matsuda H Araki N Gabapentin for Kleine-Levin syndrome Intern Med 2009 48 13 1183 1185 10.2169/internalmedicine.48.2204 19571456

[B16] Dauvilliers Y Mayer G Lecendreux M Neidhart E Peraita-Adrados R Sonka K Billiard M Tafti M Kleine-Levin syndrome: an autoimmune hypothesis based on clinical and genetic analyses Neurology 2002 59 11 1739 1745 10.1212/01.WNL.0000036605.89977.D0 12473762

[B17] Arnulf I Lin L Gadoth N File J Lecendreux M Franco P Zeitzer J Lo B Faraco JH Mignot E Kleine-Levin syndrome: a systematic study of 108 patients Ann Neurol 2008 63 4 482 493 10.1002/ana.21333 18438947

[B18] Han F Lin L Warby SC Faraco J Li J Dong SX An P Zhao L Wang LH Li QY Narcolepsy onset is seasonal and increased following the 2009 H1N1 pandemic in China Ann Neurol 2011 70 3 410 417 10.1002/ana.22587 21866560

[B19] Peraita-Adrados R Vicario JL Tafti M de León MG Billiard M Monozygotic Twins Affected with Kleine-Levin Syndrome Sleep 2012 in press 10.5665/sleep.1808 PMC3321417 22547884

